# The executive pay gap and stock price crash risk: Promotion or suppression?

**DOI:** 10.3389/fpsyg.2022.913082

**Published:** 2023-01-06

**Authors:** Qi Liu, Zicheng Pan

**Affiliations:** ^1^School of Economics and Management, Southeast University, Nanjing, China; ^2^Business School, Nanjing Normal University, Nanjing, China

**Keywords:** stock price crash risk, U-shaped relationship, information disclosure, executives, pay gap

## Abstract

**Background:**

In recent years, cases of stock price crash have continued to emerge. However, yet little research to date has investigated the compensation incentives of top management team (TMT) affect the risk of stock price crash. Nor has research considered the impact of the executive pay gap on the stock price crash risk. Especially, as the “egalitarianism” was broken in the compensation system, and the increase of the degree of marketization of salaries, the executive pay gap has shown an expanding trend. Under this circumstance, we would systematically examine the association between the extent of executive pay gap and its future stock price crash risk.

**Design, methodology, and approach:**

Based on the sample of A-Share non-financial listed companies in Shanghai and Shenzhen Stock Exchange, we used firm FE regression method to empirically examine the relationship of the internal and external compensation gaps of executives and crash risk, as well as its contigency variables and inner mechanism.

**Findings:**

The empirical results show that there is a U-shaped relationship between the internal and external pay gap of executives and future crash risk. After passing the endogenous test and the robustness test, the conclusion still holds. Further research shows that the U-shaped relationship between the pay gap and crash risk is more pronounced, when firms are affiliated with the non-state-owned enterprise or its compensation fairness is lower. Finally, the quality of information disclosure plays a mediation effect when executive pay gap affects stock price crash risk.

**Originality and value:**

According to the economic and behavior perspectives, we explored the impact of compensation structure on stock price crash risk from the pay gap of executives for the first time, and extended the emerging literature of forecasting future stock price crash risk and executive pay gap. In addition, a key implication of our findings is that more guidance for firms is provided to design the compensation structures and to reduce stock price crash risk.

## 1. Introduction

In recent years, cases of stock price crash have continued to emerge. For instance, due to the COVID-19 pandemic in 2020, the global economic crisis has resulted in a large drop in the stock price for many countries and industries. Among them, the U.S. stock market fell more than 30% in 20 trading days, the speed and magnitude of the collapse is rare in history. There is no denying that firm’s crash risk will not only erode the wealth of investors, but also damage the health of financial markets (Liu et al., 2021; Zhang et al., 2022).

In view of the significant negative impact of stock price crash risk, scholars focus on internal governance factors such as CEO equity incentives (Kim et al., 2011), excess perks (Xu et al., 2014), CEO power (Shahab et al., 2020), board diversity (Jebran et al., 2020), and information environment factors such as opaque financial reports (Hutton et al., 2009), media coverage (An et al., 2020) and ESG rating (Feng et al., 2021) to examine the cause of stock price crash risk. The basic consensus reached by the studies is that under the condition of asymmetric information, the principal-agent conflict caused by management’s self-interest, namely the management’s bad news concealing, is the root cause of stock price crash risk. To put it simply, for reasons such as compensation contracts, career and reputation concerns, and empire building (LaFond and Watts, 2008; Kothari et al., 2009), management will report good news about corporate information but not the bad. The asymmetric disclosure of good and bad news will cause negative information to be concealed inside the corporate. However, truth will come to light sooner or later. The concealed bad information will eventually be released in a concentrated way, causing the stock price to crash (Hutton et al., 2009; Shahab et al., 2020).

Based on the hypothesis of bad news concealing, compensation incentive is an important inducement for the management layer to conceal information and then trigger the stock price crash. However, the related literature is limited to CEO or CFO compensation (Kim et al., 2011; He, 2015). Few studies have directly investigated how the compensation incentives of top management team (TMT) affect the risk of stock price crash. The reason is that in addition to the CEO and CFO, there are other executives in TMT. They have the dual roles of pivotal makers and implementers and are the backbone of the corporates, whose role value is no less than that of the core executives (Lei et al., 2019). Moreover, the interaction process (such as communication, supervision, alliance) within TMT is also an important way to influence the corporate decision (Cannella et al., 2009). Therefore, it is necessary to comprehensively consider the relationship between the compensation contract of executives and stock price crash risk. Following this logic, the executive pay gap is an important part of the senior management team’s compensation contract, and its impact on the stock price crash risk cannot be ignored. Especially, as the “egalitarianism” was broken in the compensation system, and the increase of the degree of marketization of salaries, the executive pay gap has shown an expanding trend. The government has to promulgate multiple “pay restrictions” to curb the excessive compensation of some executives (Shao et al., 2021). Under this circumstance, the executive pay gap undoubtedly has a more significant impact on management’s behavior and stock price crash risk.^1^

Existing research shows that the impacts of the executive pay gap on senior managers behavior are not consistent. Specifically, from economic perspective, some scholars believe that based on Tournament Theory and Manager Market Theory, the internal and external compensation gaps of executives can alleviate the short-sightedness of the management, reduce the cost of agency, and motivate the “competitors” to work (Lazear and Rosen, 1981). From behavior perspective, some scholars believe that based on Social Comparison Theory, the internal and external compensation gap of executives will lead to dissatisfaction of underpaid executives. They may alleviate their perceived injustice through laziness, passive cooperation, and opportunistic behavior (Shaw, 2014). Thus, the executive pay gap may have both positive and negative effects on firm decision behavior. As we know, managers’ bad news concealing is an important cause of the stock price crash. So how does the executive pay gap may affect stock price crash risk through senior management behavior? In other words, is the executive pay gap suppressing the opportunistic behavior of the TMT, thereby reducing the stock price crash risk? Or is it exacerbating the opportunistic behavior, thereby increasing the stock price crash risk? This is an important issue to be addressed in this paper.

In order to answer the questions above, we use China’s A-share listed firms from 2010 to 2020 as the research samples to systematically examine the inherent relationship between the executive pay gap and stock price crash risk based on the economic and behavior perspectives. First, the internal and external pay gaps of executives both have U-shaped relationships with stock price crash risks. After passing the endogenous and the robustness test, the conclusion still holds. Second, when the corporate is a non-state-owned corporate or the compensation fairness is lower, the above U-shaped relationship is more obvious. Finally, the quality of information disclosure is a path through which the executive pay gap affects stock price crash risk.

Our paper contributes to literature in at least two ways. First, this paper extends the emerging literature of forecasting future stock price crash risk. On one side, scholars have devoted increasing attention to discussing determinant factors of future firm-specific crash risk from multiple perspectives, but few researchers focus on how executive compensation affects the risk of stock price crash. The related literature focuses on the individual level of core manager such as CEO (Kim et al., 2011; He, 2015), and few people pay attention to the impact of management team. Therefore, we have broadened the research boundaries on stock price crash risk to a certain extent and filled the deficiencies of previous studies. On the other side, existing researches have highlighted the impact of compensation on the stock price crash. We explored the impact of compensation structure on stock price crash risk from the pay gap of executives for the first time. This paper is a useful supplement to the current literature which focuses on the amount of compensation and relatively ignores the structure of compensation.

Second, this paper enriches the research on the executive pay gap. On one side, the current literature focuses on the economic consequences of the pay gap from the perspective of firm performance, R&D innovation, and executive departure (Hou, 2018; Amore and Failla, 2020; Rouen, 2020). We analyze the impact of compensation gap of senior management team based on stock price crashes from economic and behavior perspective. This not only enriches the research on compensation gap, but also reconciles the inconsistencies in the research findings on the economic consequences of compensation gap. On the other side, mainstream research generally splits the internal and external pay gap and discusses its economic consequences in a single dimension. We have built an overall thinking framework that incorporates internal and external compensation gaps into the same research system. Our research enriches the research idea of compensation gap, and has implications for comprehensively study the executive pay gap.

The remainder of the paper is structured as follows. Section 2 “Literature review and hypotheses development” reviews the related literature and presents our central predictions. Section 3 “Research design” describes our sample and research design. Sections 4 “Empirical results and 5 Expansive research” discuss the empirical results. Section 6 “Conclusion” presents our conclusion.

## 2. Literature review and hypotheses development

### 2.1. Literature review

#### 2.1.1. Stock price crash risk

Regarding the causes of future stock price crash risk, the bad news hoarding theory has been widely recognized by the academia (Jin and Myers, 2006). Scholars believe that the managerial tendency to withhold bad news leads to bad news being stockpiled within the firm. Specifically, considering a variety of managerial incentives, such as compensation contracts, career and reputation concerns, and empire building (LaFond and Watts, 2008; Kothari et al., 2009), executives have incentives to manipulate information, which is shown to propagandize the good news and conceal bad news. The asymmetric disclosure of good and bad news keeps bad information concealed inside the corporate (Hutton et al., 2009). When such a tipping point arrives, all the hitherto hidden bad news will come out at once, resulting in a large negative price adjustment, that is, the stock price crash. Subsequent scholars follow the inherent mechanism of the bad news concealing, and investigate the determinants of firm crash risk. After reviewing literature, we find that part of the scholars mainly starts from corporate internal governance, examining the impact of excess perks (Xu et al., 2014), CEO power (Shahab et al., 2020), board diversity (Jebran et al., 2020) and independent directors (Jin et al., 2022) on stock price crash. Part of scholars start with the information environment, examining the impact of financial information transparency (Hutton et al., 2009), analyst reports (Crawford et al., 2012), media coverage (An et al., 2020), and ESG rating (Feng et al., 2021) on crash risk. Another part of the scholars starts with the manager characteristics, examining the impact of CEO power (Shahab et al., 2020), CEO overconfidence (Kim et al., 2016), and top executive gender (Li and Zeng, 2019) on crash risk. To sum up, although the contributing factors of stock price crashes are different, the inherent mechanism is that managers hide and accumulate bad news by manipulating information and using other opportunistic behavior under asymmetric information.

#### 2.1.2. Executive pay gap

The TMT is the important human capital of the corporate. How to motivate senior managers to work hard and reduce the agency conflict is always an important issue. Among them, executive compensation incentive is the most widely used method, and the pay gap of top executives^2^ is an important part of compensation incentive, which reflects efficiency and fairness to some extent. However, the debate in academia over whether the pay gap incentive is effective is still endless, and no consensus has yet been reached.

Researchers from economic perspective believe that compensation incentives are analogous to sports championships based on Tournament Theory. Senior managers are like competitors in a tournament. Winners obtain promotions and huge bonuses, while losers get nothing. As a result, internal pay gap can encourage executives to work hard, and consequently to improve corporate performance (Lazear and Rosen, 1981; Faleye et al., 2013). The bulk of the studies support the tournament theory. For example, based on the empirical evidence of Chinese listed companies, Lin and Lu (2009) show that internal compensation gap (INGAP) can promote corporate performance. Vieito (2012) finds that a higher internal pay gap between CEO and TMT leads to higher corporate performance. As identified by Ridge et al. (2015), the larger the INGAP of executives, the lower the non-CEO turnover rate will be. In contrast, researchers from behavior perspective believe that, according to social comparison theory, executives will subconsciously compare their output-input ratio with selected reference objects horizontally or vertically to judge the fairness of their compensation (Adams, 1965). When the INGAP of executives is too large, the underpaid managers will feel oppressed. In this case, the willingness and satisfaction of the executives to cooperate will decrease, which will bring negative impact on the enterprise (Shaw, 2014). For example, Carpenter and Sanders (2002) find that CEO-TMT pay gap has a significant negative impact on the corporate performance. Bloom and Michel (2002) point out that vertical pay disparity between top managers-middle managers is significantly negatively related to the average tenure of senior managers.

With the continuous improvement of the marketization of pay and managers turnover velocity, scholars have devoted increasing attention to the phenomenon of external salary gap incentive. The external compensation gap reflects certain social distribution characteristics and is an important reference for executives to perceive the amount of compensation (Gao, 2019). Like INGAP, the academia has not yet obtained a clear understanding of whether it is effective in increasing the external compensation gap. According to social comparison theory and reference point theory, scholars from the behavioral perspective deem that excessive external pay gap brings negative emotions to underpaid managers, triggering slack work and negative cooperation. This will lead to a decline in the corporate performance and increase the possibility of voluntary departures and duty-related crimes (Faulkender and Yang, 2010). However, according to the manager market theory, researchers from economic perspective point out that in an effective manager market, external compensation gap between executive compensation and peers’ compensation benchmarks can drive low-paid executives to try their best to change their disadvantaged position. It can also motivate high-paid executives to work hard to cope with competitive pressures, thereby improving corporate performance (Lin and Lu, 2009). In addition, some researchers believe that the external compensation gap and corporate performance is not just a linear relationship, and there is an interval effect between them (Gao, 2019).

#### 2.1.3. Deficiencies in current research

Combing through literature, it is found that there are deficiencies in current research. First, although the existing literature discusses the determinants of stock price crash risk from multiple aspects, it does not pay attention to the compensation incentives that induce managers to conceal bad news, and the pay structure is barely mentioned. Second, most literature concentrates on the incentive effects of compensation gap, such as corporate performance, innovation investment, and manager departures. However, there is very little literature examining the economic consequences of compensation gap from the perspective of market risk. Third, most scholars split the INGAP from the external compensation gap and discuss their economic consequences unilaterally. However, executives will perform a horizontal and vertical comparisons to perceive compensation contracts, thus implementing behavioral decisions. Moreover, although there are overlaps in internal and external compensation gaps, there are many differences in intrinsic mechanism. Therefore, it is necessary to conduct a comparative study of internal and external pay gaps at the same time. Forth, academia has not yet reached a consensus on the consequences of the executive compensation gap. These inconsistent and even contradictory conclusions indicate that the academia needs to further explore the inherent logic of compensation gap.

### 2.2. Hypotheses development

Through literature review, it is easy to find that the existing research on the pay gap is mainly based on economic and behavior perspectives. The economic perspective emphasizes competition, focusing on the positive effects of the pay gap. The behavior perspective emphasizes cooperation, focusing on its negative effects. We believe that as the compensation gap continues to increase, the two perspectives of competition and cooperation may play a leading role at different stages. Therefore, its impact on stock price crash risk is also an evolving process. Considering that executives will refer to the internal and external pay gap to perceive compensation contracts, we will combine two perspectives to analyze the inherent relationship from between pay gap and stock price crash risk from the two aspects of internal and external pay gap.

#### 2.2.1. Internal pay gap of executives and stock price crash risk

When the internal pay gap is within a moderate range, a reasonable compensation level can provide enough incentives for senior management team. Therefore, the pay gap is more a reflection of competition. In this phase, the tournament theory from economic perspective plays a leading role, and the internal pay gap of executives contributes to reducing the crash risk. There are three reasons for this. First, based on tournament theory, the modest compensation gap between the tournament winner and the remaining players can create a competitive organizational climate, and motivate executives to exert higher effort and align the interest between principals and agents. They in turn can mitigate agency problems and curb executive misbehavior (Lazear and Rosen, 1981). Second, the TMT is an inherent constraint mechanism, and executives can exercise their supervisory powers with each other (Cheng et al., 2016). Especially motivated by compensation tournaments, executive members who compete and understand each other pay attention to each other. If provision of false financial reports, concealment of negative information and other illegal behaviors are learned by competitors, the executives are likely to be “out” in this tournament. As a result, short-termist behaviors will be curbed, and the quality of information disclosure will be improved accordingly. Third, the appropriate pay gap can reduce conspiracy among executives. The earnings manipulation, financial fraud, etc. are collective behaviors of multiple executives, not individual behaviors. However, the tournament-style compensation incentive can make executives grudge. This psychological distance will weaken the collusion among executives, and reduce self-interest behaviors such as bad news concealing. In view of this, we conjecture that an opportune internal pay gap of executives enables executives to reduce managerial opportunism and improve the quality of information disclosure, ultimately leading to the decrease of crash risk.

However, when the internal pay gap exceeds the threshold, excessive pay gap would destroy a positive competition, and increase the dissatisfaction and inequality of lower-level management. In this phase, the theory from behavior perspective occupies a leading role, and the internal pay gap will increase future crash risk. The reason is as follows. On the one hand, in a social comparison theory framework, excessive internal pay gap enables lower-level managers feel that their input and outcome tradeoffs are far inferior to those of executives, and perceive a sense of inequality and deprivation (Lin and Lu, 2009). These negative perceptions can cause them to decrease their work efforts, engage in more earnings manipulation and outright resource diversion, and thus aggravate the principal-agent conflict (Shaw, 2014). Kini and Williams (2012) find that large compensation gap between core executives and lower-level executives have enticed lower-level managers to engage in riskier policies in order to reverse unfairness. It suggests that the excessive pay gap within TMT not only fails to produce incentive-oriented effect but also reduces the quality of information disclosure. On the other hand, social comparison theory believes that the unfairness caused by the huge compensation gap is because lower-level executives do not reach their psychological expectations and thus feel excessive compensation losses. Combined with the prospect theory “loss preference risk” view (Kahneman and Tversky, 1979), lower-level management will invest blindly. In order to advance to a higher hierarchy, they often underestimate the risks and increase the high-risk investments scale (Kini and Williams, 2012). However, to continue with a risky investment project, executives will not disclose the investment information in a timely and truthful manner. While deteriorating the corporate information environment, it enables shareholders and regulators to fail to prevent negative NPV investment projects due to lack of effective information, causing a large amount of accumulated bad news and a large stock price drop. Thus, the excessive internal pay gap of executives may result in a price crash. We propose Hypothesis 1a:

*Hypothesis 1a: There is a U-shaped relationship between the internal pay gap of executives and stock price crash risk.*

#### 2.2.2. External pay gap of executives and stock price crash risk

Similarly, when the external pay gap between executive compensation and peers’ compensation benchmarks is within a moderate range, relatively flowing executives will compete in order to cultivate reputation and obtain high compensation. In this phase, the manager market theory from economic perspective will play a leading role, and the crash risk will decrease as the external pay gap increases. Specifically, first, based on manager market theory, when external pay gap is within a moderate range, senior executives with lower compensation can exert more effort and ease the conflict of the agent (Gu and Yang, 2018). On the one hand, they can seek higher compensation through a free-flowing manager market. On the other hand, they can also put pressure on the board of directors to receive a higher compensation. To deal with competition pressure from executives, senior executives with higher compensation will expend more time and energy in work in a bid to build a good reputation (Li et al., 2014). Consequently, executives will abandon behaviors such as earnings manipulation and bad news hoarding that may bring reputation costs, which can alleviate the principal-agent conflict and improve the corporate information quality. Second, based on manager talent signal hypothesis, in order to respond to the doubts about the high compensation of senior executives and reduce the possible “anger cost,” higher-paying executives have the incentive to actively disclose information (business plan or strategic information) to demonstrate their own talents (Baik et al., 2011). This can confirm the “legality” and “legitimacy” of compensation to the outside world, and help defend for high compensation. In conclusion, the expanding external pay gap within a moderate range enable executives to alleviate the principal-agent conflict and improve the quality of information disclosure, which, in turn, the bad news hoarding will be curbed and the crash risk will be reduced accordingly.

However, as the external compensation gap widens, the excessive pay gap enables higher-paying executives to induce psychological bias of overconfidence. In this phase, theories from behavior perspective play a key role, and the probability of crash risk increases with the external compensation gap. Specifically, in addition to the compensation of human capital, the huge gap in executive compensation will also bring positive feedback to the ability and self-importance of executives. Combined with the theory of self-attribution, the positive feedback that executive compensation is higher than peer-reviewed salary benchmark can enhance managerial confidence and self-esteem, and then stimulate a psychological bias of overconfidence (Wen and Tang, 2012). Further research shows that overconfident executives tend to boast their risk-taking incentives, tending to overestimate the returns and underestimate the risk of investment, leading to over-investment (Malmendier et al., 2011). For investment to be implemented smoothly, executives often deliberately withhold and accumulate negative information. As the investment continues to operate, bad news hoarding eventually reaches a tipping point and is disclosed to the market, thus leading to a stock price crash. Moreover, studies have shown that overconfident managers have a tendency to provide incorrect earnings forecasts and withhold bad news (Hsieh et al., 2014), leading to a large negative drop in stock price. We propose Hypothesis 1b:

*Hypothesis 1b: There is a U-shaped relationship between the external compensation gap of executives and stock price crash risk.*

## 3. Research design

### 3.1. Sample selection

Considering the availability of data, this study selects A-share listed companies in Shanghai Stock Exchange and Shenzhen Stock Exchange from 2010 to 2020 as initial samples. According to Cheng et al. (2021) and Xu et al. (2014), we filtered out the preliminary sample according to the following criteria. (1) ST or PT type listed enterprises were excluded. (2) Sample companies lacking important data were excluded. (3) Financial services firms were excluded. (4) Firms with fewer than 30 weeks of stock return data were excluded. (5) Sample companies of undisclosed and zero CEO compensation were excluded. Our final sample comprises unbalanced panel data of 3,800 companies that generate 28,505 observations from 2010 to 2020. We retrieved corporate governance, firm-level accounting information, and compensation data of senior management team data from the CSMAR database. The data to calculate risk of stock price crash were also obtained from the Wind database. In addition, we also checked the financial statements and information announcements for suspicious data. To eliminate the influence of extreme values, we winsorize all continuous variables all scaled variables at the top and bottom 1% of each distribution.

### 3.2. Variable definition

#### 3.2.1. Risk of stock price crash

Referring to Kim et al. (2011) and Cheng et al. (2021), we use the following methods to measure the risk of stock price crash of listed companies. We first estimate firm-specific weekly returns, denoted W, as the natural log of one plus the residual return from the expanded market model regression for each firm and year:


$${\text{R}}_{i,k}=\alpha_{\text{i}}+\gamma_{1}\,{\text{R}}_{m,k-2}+\gamma_{2}\,{\text{R}}_{m,k-1}+\gamma_{3}\,{\text{R}}_{m,k}+\gamma_{4}\,{\text{R}}_{m,k+1}$$

(1) $$+\gamma_{5}\,{\text{R}}_{i,k+2}+\delta_{i,k}$$

where R_i,k_ is the return on stock i in week k, and R_m,k_ is the value-weighted A-share market return in week *t*. The firm-specific weekly return for firm i in week *t*, W_*i,t*_ is measured by the natural log of one plus the residual return in Equation (1), that is, W_*i*,t_ = Ln (1 + δ _*i,k*_).

The first measure of crash risk is the negative conditional return skewness (NCSKEW). NCSKEW is calculated by taking the negative of the third moment of firm-specific weekly returns for each year and normalizing it by the standard deviation of firm-specific weekly returns raised to the third power. Specifically, for each firm i in year *t*, NCSKEW is calculated as:


$${\text{NCSKEW}}_{i,t}=-\left[n\times {\left(\text{n}-1\right)}^{3/2}\times \sum {\text{W}}_{i,t}^{3}\right]/[\left(n-1\right)$$

(2) $$\times \left(\text{n}-2\right)\times {\left(\sum {\text{W}}_{i,t}^{2}\right)}^{3/2}]$$

where *n* is the number of observations of firm-specific weekly returns of firm i during year t, 30≦ *n*≦52.

The second measure of crash risk is down-to-up volatility, denoted as DUVOL, which captures the asymmetric volatility between negative and positive firm-specific weekly return. For firm i in year t, we separate all the weeks with firm-specific weekly returns below the annual mean (down weeks) from those with firm-specific returns above the annual mean (up weeks) and calculate the standard deviation for each of these subsamples separately. We then take the log of the ratio of the standard deviation of the down weeks to the standard deviation of the up weeks. Expressed mathematically,


$${\text{DUVOL}}_{i,t}=-\text{Ln}\{\;\left[\left({\text{n}}_{\text{u}}-1\right)\times \sum_{\text{down}}{\text{W}}_{i,t}^{2}\right]/[\left({\text{n}}_{\text{d}}-1\right)$$

(3) $$\;\times \sum_{\text{up}}{\text{W}}_{i,t}^{2}]\}$$

where n_*u*_ and n_*d*_ are the numbers of up and down weeks, respectively.

#### 3.2.2. Compensation gap of senior management team

Regarding the INGAP, we refer to Faleye et al. (2013) and define the INGAP of the senior management team as the compensation ratio between core and non-core managers. Core manager compensation is measured by the average of the top three managers’ compensations. Non-core managers’ compensations are measured by total managers’ compensation minus the top three managers’ compensation, divided by the number of remaining managers (excluding the number of unpaid managers). Regarding the external compensation gap (EXGAP), we refer to Gu and Yang (2018) and take the ratio of the average compensation of senior management team in this corporate to the average compensation of senior management team in the same industry as a measure of external compensation gap.

#### 3.2.3. Control variables

To isolate the effect of conservatism on crash risk from the effects of other variables, we include several control variables known to influence crash likelihood. Following Kim et al. (2011) and Chen et al. (2021), we include a set of control variables deemed to be potential predictors of crash risk. The variable HSL is the detrended stock trading volume, which is a proxy for investor heterogeneity, or the difference in opinions between investors. The lagged NCSKEW variable is the negative skewness of past firm-specific stock returns, which is included to capture the potential persistence of the third moment of stock returns. The variable SIGMA is the standard deviation of past firm-specific stock weekly returns, and RET is the average firm-specific weekly return over the past year. Following Chen et al. (2001) and Xu et al. (2014), we also include standard control variables such as SIZE, defined as the logarithm of a firm’s total assets; MB, defined as the ratio of the market value of equity to the book value of equity; LEV, defined as the book value of all liabilities scaled by the book value of assets; and ROA, defined as the income before extraordinary items divided by total assets; ABACC, defined as discretionary accruals as estimated from the modified Jones model. As identified by Kim et al. (2011) and Wen et al. (2020), a CEO’s equity ownership and institutional investors may affect his or her incentive to withhold bad news and hence is associated with future crash risk. To control for this incentive, we include CEO_SHARE, defined as the percentage of shares held by the CEO; INST, defined as the percentage of shares held by the institutional investors. In all regressions, we also include industry and fiscal year indicators to control for industry and time fixed effects (FE). Table 1 lists and defines the variables used in the paper.

**TABLE 1:** Definition of each variable.

Meaning	Declaration
**Dependent variable (T+1)**	

*NCSKE*	The negative coefficient of skewness, calculated by taking the negative of the third moment of firm-specific weekly returns for each sample year and dividing it by the standard deviation of firm-specific weekly returns raised to the third power. See Equation (3) for details
*DUVOL*	The down-to-up volatility. For any stock i in year t, we separate all the weeks with firm-specific weekly returns below the annual mean (down weeks) from those with firm-specific weekly returns above the period mean (up weeks) and compute the standard deviation for each of these subsamples separately. We then take the log of the ratio of the standard deviation of the down weeks to the standard deviation of the up weeks. See Equation (4) for details

**Independent variables (*t*)**	

INGAP	Average compensation of core managers divided by average compensation of non-core managers
EXGAP	Average compensation of senior management team in target corporate divided by average compensation of senior management team in the same industry

**Control variables (t)**	

RET	The mean of firm-specific weekly returns over the fiscal year, times 100
*SIGMA*	The standard deviation of firm-specific weekly returns over the fiscal year
HSL	The average monthly share turnover for the current fiscal year minus the average monthly share turnover for the previous fiscal year
*ROA*	Firm profitability, calculated as income before extraordinary items divided by total assets
*SIZE*	The log of the firm’s total assets
*LEV*	The book value of all liabilities scaled by the book value of assets
*MB*	The market-to-book ratio
INST	The percentage of outstanding shares owned by institutional investors
CEO_SHARE	The percentage of outstanding shares owned by a firm’s CEO
ABACC	The residuals from the modified Jones model

### 3.3. Model design

To investigate the impact of compensation gap of TMT on stock price crash risk, the following regression model was constructed:


CRASHi,t+1=γ0+γ1⁢PAYGAPi,t+γ2⁢PAYGAPi,t2

$$+\gamma_{3}\,{\text{CRASH}}_{i,t}+\gamma_{4}\,{\text{RET}}_{i,t}+\gamma_{5}\,{\text{SIGMA}}_{i,t}$$

$$+\gamma_{6}\,{\text{HSL}}_{i,t}+\gamma_{7}\,{\text{ROA}}_{i,t}+\gamma_{8}\,{\text{SIZE}}_{i,t}$$

$$+\gamma_{9}\,{\text{LEV}}_{i,t}+\gamma_{10}\,{\text{MB}}_{i,t}+\gamma_{11}\,{\text{INST}}_{i,t}$$

$$+\gamma_{12}\,{\text{MGR}}_{i,t}+\gamma_{13}\,{\text{ABACC}}_{i,t}+\gamma_{14}\,{\text{AUDIT}}_{i,t}$$

$$+\gamma_{15}\,{\text{AFN}}_{i,t}+\mathrm{Year}\,\mathrm{Dummies}$$

(4) $$+\mathrm{Industry}\,\mathrm{Dummies}+\varepsilon_{i,t}$$

where the dependent variable, CRASh is proxied by NCSKEW or DUVOL. Our primary independent variable, PAYGAP is proxied by INGAP and EXGAP. The term CV represents the set of control variables as discussed above. The dependent variable is measured in year t+1, while the independent variables and control variables are measured in year t. The above regressions control for year and industry FEs. Regression equations are estimated using firm FE with White standard errors corrected for firm clustering.^3^ If the coefficient γ_*1*_ and γ_*2*_ corresponding to PAYGAP and its square term are significantly non-zero, the relationship between internal and external compensation gap and stock price crash risk can be determined according to the coefficient values of γ_*1*_ and γ_*2*_. If γ_*1*_ > 0, γ_*2*_ < 0, there is an inverted U-shaped curve between the pay gap and the stock price crash risk. If γ_*1*_ < 0, γ_*2*_ > 0, there is a U-shaped curve between the pay gap and the stock price crash risk.

## 4. Empirical results

### 4.1. Descriptive statistics

Table 2 shows descriptive statistics of main variables. The average (median) of NCSKEW_i,t+1_ and DUVOL_i,t+1_ are –0.321 (–0.273) and –0.212 (–0.215), respectively. This is similar to the findings of Cheng et al. (2021). The average value of INGAP_i,t_ is 6.804, indicating that core managers compensation is 6.804 times that of non-core managers. The average value of EXGAP_i,t_ is 0.984, indicating that the average managers compensation of listed companies is 0.984 times that of the peer-reviewed salary benchmark. The extreme difference in internal and external compensation is relatively large, indicating that the compensation gap between different corporates is more obvious.

**TABLE 2:** Descriptive statistics of the main variables.

Variables	Observations	Average	Standard deviation	Median	Minimum	Maximum
NCSKEW_i,t + 1_	28,505	–0.321	0.722	–0.273	–2.426	1.696
DUVOL_i,t + 1_	28,505	–0.212	0.475	–0.215	–1.353	1.050
INGAP_i,t_	28,505	6.804	5.782	4.984	2.075	38.454
EXGAP_i,t_	28,505	0.984	0.747	0.779	0.137	4.640
NCSKEW_i,t_	28,505	–0.316	0.725	–0.276	–2.416	1.715
RET_i,t_	28,505	0.003	0.010	0.002	–0.016	0.038
SIGMA_i,t_	28,505	0.064	0.026	0.058	0.026	0.162
HSL_i,t_	28,505	–0.120	0.464	–0.064	–2.004	0.978
ROA_i,t_	28,505	0.035	0.069	0.036	–0.388	0.212
SIZE_i,t_	28,505	22.169	1.311	21.990	19.114	27.050
LEV_i,t_	28,505	0.429	0.211	0.421	0.053	1.157
MB_i,t_	28,505	0.975	0.990	0.695	0.101	6.013
INST_i,t_	28,505	0.439	0.245	0.458	0.003	0.905
MGR_i,t_	28,505	0.049	11.123	0.000	0.000	0.510
ABACC_i,t_	28,505	0.070	0.073	0.049	0.001	0.424

### 4.2. Multiple regression analysis

Columns (1) and (2) of Table 3 are the results of multiple regressions of the internal pay gap of executives and stock price crash risk. The results show that after controlling related factors, when NCSKEW_i,t+1_ is used to measure future crash risk, the regression coefficient of INGAP_i,t_ is significantly negative, and the regression coefficient of INGAPi,t2 is significantly positive.

**TABLE 3:** Regression analysis to pay gap and stock price crash risk.

	NCSKEW_i,t+ 1_	DUVOL_i,t+ 1_	NCSKEW_i,t+ 1_	DUVOL_i,t+ 1_
				
	(1)	(2)	(3)	(4)
INGAP_i,t_	–0.0036**	–0.0025**		
	(–2.03)	(–2.17)		
INGAPi,t2	0.0002***	0.0001**		
	(2.67)	(2.22)		
EXGAP_i,t_			–0.0490**	–0.0315**
			(–2.41)	(–2.33)
EXGAPi,t2			0.0211***	0.0158***
			(3.79)	(4.20)
NCSKEW_i,t_	–0.0772***	–0.0410***	–0.0771***	–0.0409***
	(–12.15)	(–9.98)	(–12.15)	(–9.97)
RET_i,t_	8.2042***	5.5567***	8.1635***	5.5252***
	(11.99)	(12.14)	(11.93)	(12.07)
SIGMA_i,t_	–0.8638***	–0.7664***	–0.8531***	–0.7608***
	(–2.97)	(–3.99)	(–2.93)	(–3.96)
HSL_i,t_	0.0051	0.0046	0.0047	0.0043
	(0.50)	(0.69)	(0.46)	(0.64)
ROA_i,t_	–0.4217***	–0.2980***	–0.4261***	–0.3025***
	(–4.71)	(–5.06)	(–4.75)	(–5.11)
SIZE_i,t_	0.0290**	0.0013	0.0307**	0.0014
	(2.38)	(0.16)	(2.46)	(0.17)
LEV_i,t_	–0.0948**	–0.0488	–0.0987**	–0.0517*
	(–2.04)	(–1.60)	(–2.12)	(–1.70)
MB_i,t_	–0.0687***	–0.0419***	–0.0681***	–0.0411***
	(–7.27)	(–6.93)	(–7.20)	(–6.80)
INST_i,t_	0.3668***	0.2394***	0.3668***	0.2381***
	(7.41)	(7.39)	(7.41)	(7.35)
MGR_i,t_	0.0012	0.0009*	0.0013	0.0009*
	(1.55)	(1.65)	(1.63)	(1.73)
ABACC_i,t_	0.1384**	0.0999**	0.1359**	0.0978**
	(2.18)	(2.38)	(2.14)	(2.33)
Constant	–1.0024***	–0.1786	–1.0062***	–0.1533
	(–2.98)	(–0.87)	(–2.95)	(–0.73)
YEAR_i,t_/IND_i,t_	Control	Control	Control	Control
*N*	28,505	28,505	28,505	28,505
*R* ^2^	0.049	0.048	0.049	0.049

This table presents the fixed effect regression results of the impact of the pay gap of executive on stock price crash risk. The sample contains firm-years from 1995 to 2008 with non-missing values for all the control variables. Year and industry fixed effects are included. The t-statistics reported in parentheses are based on standard errors clustered by both firm and year. *, **, and ***Indicate statistical significance at the 10, 5, and 1% levels, respectively.

**FIGURE 1:**
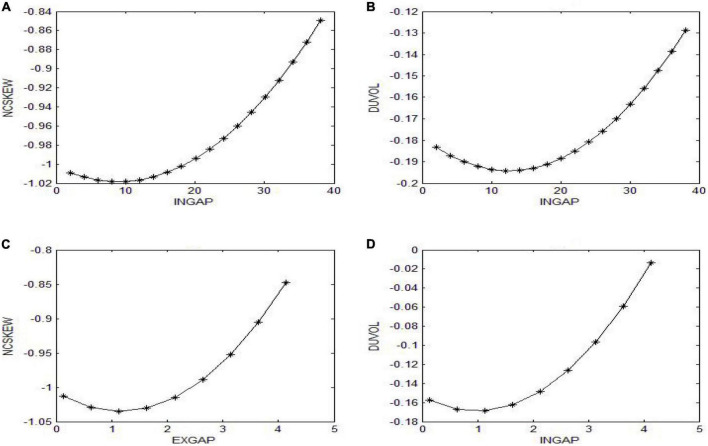
U-shaped curve of the pay gap and stock price crash risk. **(A)** U-shaped curve of the INGAP and NCSKEW. **(B)** U-shaped curve of the INGAP and DUVOL. **(C)** U-shaped curve of the EXGAP and NCSKEW. **(D)** U-shaped curve of the EXGAP and DUVOL.

Similarly, when DUVOL_i,*t1*_ is used to measure future crash risk, the regression coefficient of INGAP_i,t_ is significantly negative, and the regression coefficient of INGAPi,t2 is significantly positive. See Figures 1A, B for the specific relationship. These results suggest there is a U-shaped relationship between the internal compensation gap and future crash risk. Hypothesis 1a is verified. Columns (3) and (4) of Table 3 are the results of multiple regressions of the external compensation gap of senior management team and the risk of stock price crash. The results show that after controlling related factors, when NCSKEW_i,*t1*_ and *DUVO*L_i,*t1*_ are used to measure the risk of stock price crash, the regression coefficient of EXGAP_i,t_ is significantly negative, and the regression coefficient of EXGAPi,t2 is significantly positive. See Figures 1C, D for the specific relationship. This suggests a U-shaped relationship between the external compensation gap and future crash risk. Hypothesis 1b is verified.

In addition, the inflection points^4^ of U-shaped curve as shown in column (1) and column (2) are 9.347 and 12.536, respectively. In the whole sample, the 75 and 90% quantile of the internal pay gap is 8.503 and 12.5041, respectively. The inflection points of U-shaped curve as shown in column (3) and column (4) are 1.1677 and 0.996, respectively. In the entire sample, the 65 and 80% quantile of the external pay gap is 0.998 and 1.204, respectively. These result shows that most firms are in the falling stage of the U-shaped curve, and only a small number of firms are in the rising stage of the U-shaped curve. Accordingly, it can be inferred that the setting of Chinese corporate compensation structure is relatively scientific and reasonable, which plays a positive role in curbing market risk.

### 4.3. Endogeneity analysis and robustness test

#### 4.3.1. Endogeneity analysis

There may be endogeneity problems such as reverse causality, omitted variables, and measurement biases in the research model. These may lead to biased parameter estimates. In order to avoid the interference of parameter estimation bias on the research conclusions, we employ the FE instrumental variables models (FE-IV) to estimate the model. Specifically, the first stage is the test of the compensation gap to obtain the estimated value of compensation gap. Drawing on the methods of Kini and Williams (2012), we select the following variables as instrumental variables: INASG_i,t_, measured by the internal average compensation gap in the same industry; EXASG_i,t_, measured by the external average compensation gap in the same industry; IAS_i,t_, measured by employee average salary in the same industry and province. These factors will not directly affect future crash risk, and can only affect the risk through compensation gap. Therefore, theoretically, the selected variables satisfy the requirements of the correlation and exogenous of instrumental variables. In addition to the variables above, we also add a set of control variables which are consistent with the control variables in Equation 4. In the second stage, we use the predicted value of the internal and external compensation gap in the first stage as independent variables, and perform a regression analysis again. The results are shown in Table 4.

**TABLE 4:** Regression analysis to address endogeneity concerns.

	1st stage		2nd- stage			
	INGAP_i,t_	EXGAP_i,t_	NCSKEW_i,t+ 1_	DUVOL_i,t+ 1_	NCSKEW_i,t+ 1_	DUVOL_i,t+ 1_
						
	(1)	(2)	(3)	(4)	(5)	(6)
INGAP_i,t_			–0.1835***	–0.1134***		
			(–5.40)	(–5.11)		
INGAPi,t2			0.0241***	0.0133***		
			(5.03)	(4.27)		
EXGAP_i,t_					–0.1282**	–0.1034***
					(–2.37)	(–2.91)
EXGAPi,t2					0.0575*	0.0649***
					(1.79)	(3.04)
INASG_i,t_0.5936***						
(9.45)						EXASG_i,t_3.6105***
						(29.39)
				IAS_i,t_-0.3446**	0.1640***	
				(–2.37)	(13.70)	
			NCSKEW_i,t_	0.0391	0.0239***	–0.0786***
-0.0423***	–0.0755***	–0.0402***		(0.79)	(4.40)	(–12.34)
(–10.26)	(–11.75)	(–9.67)	RET_i,t_	8.6341	1.2901*	7.9822***
5.3460***	8.2487***	5.5466***		(1.50)	(1.90)	(11.57)
(11.59)	(12.02)	(12.09)	SIGMA_i,t_	3.6043	–0.0958	–0.8885***
-0.8317***	–0.8752***	–0.7752***		(1.61)	(–0.39)	(–3.00)
(–4.26)	(–3.01)	(–4.04)	HSL_i,t_	0.0690	0.0073	0.0050
0.0037	0.0056	0.0049		(0.83)	(0.94)	(0.49)
(0.55)	(0.55)	(0.72)	ROA_i,t_	–3.1483***	1.2381***	–0.4929***
-0.3009***	–0.2919***	–0.2001***		(–4.43)	(18.59)	(–4.94)
(–4.59)	(–2.75)	(–2.86)	SIZE_i,t_	–0.2024***	0.2714***	0.0318**
0.0056	0.0499***	0.0129		(–4.53)	(46.91)	(2.55)
(0.68)	(3.08)	(1.21)	LEV_i,t_	–0.8829***	–0.0118	–0.0807*
-0.0299	–0.0971**	–0.0508*		(–3.70)	(–0.52)	(–1.68)
(–0.95)	(–2.09)	(–1.67)	MB_i,t_	–0.1079**	–0.1512***	–0.0667***
-0.0393***	–0.0766***	–0.0443***		(–2.05)	(–20.41)	(–6.98)
(–6.43)	(–6.87)	(–6.20)	INST_i,t_	–1.1426***	0.1538***	0.3901***
0.2653***	0.3744***	0.2420***		(–6.34)	(8.05)	(7.58)
(7.89)	(7.53)	(7.45)	MGR_i,t_	0.0009	0.0004	0.0011
0.0007	0.0013	0.0009*		(0.25)	(1.37)	(1.35)
(1.44)	(1.62)	(1.71)	ABACC_i,t_	1.6558***	–0.0764	0.1321**
0.0756*	0.1318**	0.0929**		(3.07)	(–1.40)	(1.96)
(1.70)	(2.07)	(2.21)	Constant	20.2337***	–3.0403***	–2.3200***
-1.0647***	–1.3536***	–0.3457		(10.83)	(–14.92)	(–5.49)
(–4.01)	(–3.47)	(–1.42)	YEAR_i,t_/IND_i,t_	Control	Control	Control
Control	Control	Control	*N*	28,494	28,494	28,505
28,505	28,505	28,505	*R* ^2^	0.031	0.025	0.050
0.049	0.049	0.049				

This table presents the 2SLS regression results of the impact of the executive pay gap on stock price crash risk. The sample contains firm-years from 2010 to 2020 with non-missing values for all the control variables. Year and industry fixed effects are included. The *t*-statistics reported in parentheses are based on standard errors clustered by both firm and year. *, **, and ***Indicate statistical significance at the 10, 5, and 1% levels, respectively.

In column (1) of Table 4, the regression coefficient of INASG_i,t_ is significantly positive at the 1% level. The regression coefficient of IAS_i,t_ is significantly negative at the 5% level. In column (2), the regression coefficients of EXASG_i,t_ and IAS_i,t_ are significantly positive at the 1% level. In addition, INASG_i,t_, EXASG_i,t_ and IAS_i,t_ are not related to the corresponding regression residuals, indicating that the null hypothesis of “all instrumental variables are exogenous” is accepted. The selected variables can satisfy the two preconditions of relevance and exogeneity. The results in column (1)–(4) of Table 4 show that the regression coefficients of internal and external compensation gap are significantly negative and their square terms are significantly positive. It indicates that the U-shaped relation between executive pay gap and crash risk still holds after controlling for endogeneity.

#### 4.3.2. Robustness test

In order to ensure the validity of the conclusions, we conducted several additional tests to check the robustness of our results.

First, replace the pay gap variable. For INGAP, we refer to Kini and Williams (2012) and use the natural logarithm of the difference between core managers’ compensation and non-core managers’ compensation as a measurement of INGAP. For external compensation gap, we refer Li et al. (2014), and use the ratio of the managers’ average compensation and the managers’ maximum compensation in the same industry as a proxy variable for external compensation gap. We re-estimated our model using the new measure of compensation gap. Results reported in Panel A of Table 5 show that the regression coefficients for INGAPi,t2 and EXGAPi,t2 are significantly positive, indicating that the aforementioned conclusions are robust.

**TABLE 5:** Regression analysis to robustness test.

Panel A				
	NCSKEW_i,t+ 1_	DUVOL_i,t+ 1_	NCSKEW_i,t+ 1_	DUVOL_i,t+ 1_
				
	(1)	(2)	(3)	(4)
INGAP_i,t_	–0.1521**	–0.0991**		
	(–2.21)	(–2.15)		
INGAPi,t2	0.0120**	0.0078**		
	(2.32)	(2.24)		
EXGAP_i,t_			–0.1382*	–0.0556
			(–1.76)	(–1.08)
EXGAPi,t2			0.2372**	0.1571**
			(2.53)	(2.52)
Constant	1.6081*	1.3299**	–1.0863***	–0.2353
	(1.78)	(2.19)	(–3.20)	(–1.13)
YEAR_i,t_/IND_i,t_	Control	Control	Control	Control
*N*	28,460	28,460	28,466	28,466
*R* ^2^	0.047	0.050	0.049	0.048
**Panel B**				
INGAP_i,t_	–0.0061**	–0.0044**		
	(–2.06)	(–2.36)		
INGAPi,t2	0.0003***	0.0002***		
	(3.08)	(2.86)		
EXGAP_i,t_			–0.1286***	–0.0664***
			(–3.36)	(–2.66)
EXGAPi,t2			0.0370***	0.0217***
			(3.60)	(3.06)
Constant	–0.9179	0.2248	1.4182	1.4538***
	(–1.22)	(0.47)	(1.56)	(2.65)
YEAR_i,t_/IND_i,t_	Control	Control	Control	Control
*N*	13,005	13,005	13,005	13,005
*R* ^2^	0.106	0.092	0.106	0.092
**Panel C**				
INGAP_i,t_	–0.0036**	–0.0025**		
	(–2.06)	(–2.20)		
INGAPi,t2	0.0002***	0.0001**		
	(2.69)	(2.24)		
EXGAP_i,t_			–0.0494**	–0.0315**
			(–2.42)	(–2.33)
EXGAPi,t2			0.0212***	0.0158***
			(3.82)	(4.21)
DUAL_*i,t*_	–0.0158	–0.0040	–0.0206	–0.0086
	(–0.37)	(–0.14)	(–0.48)	(–0.31)
DDR_*i,t*_	–0.0065	–0.0070	–0.0051	–0.0061
	(–0.39)	(–0.64)	(–0.30)	(–0.56)
BIG4_*i*,t_	0.1677	0.1123	0.1615	0.1088
	(1.34)	(1.35)	(1.29)	(1.31)
FIRST_HOLD_*i,t*_	–0.0016**	–0.0010*	–0.0016**	–0.0010*
	(–1.98)	(–1.83)	(–2.03)	(–1.88)
Constant	–0.9791***	–0.1667	–0.9791***	–0.1395
	(–2.85)	(–0.79)	(–2.82)	(–0.66)
YEAR_i,t_/IND_i,t_	Control	Control	Control	Control
*N*	28,505	28,505	28,505	28,505
*R* ^2^	0.049	0.049	0.049	0.049

This table presents the robustness test results of the impact of the executive pay gap on stock price crash risk. The sample contains firm-years from 2010 to 2020 with non-missing values for all the control variables. The results of measurement replacement are shown in Panel A. The results of sample replacement are shown in Panel B. The results of adding control variables are shown in Panel C. To save space, we omit the coefficient estimates of the control variables (except for adding control variables in Panel C). Year and industry fixed effects are included. The t-statistics reported in parentheses are based on standard errors clustered by both firm and year. *, **, and ***Indicate statistical significance at the 10, 5, and 1% levels, respectively.

Second, replace sample. In mid-2015, China experienced an extremely severe stock market crash. Thousands of stocks hit the limit-down, and the impact was far-reaching. In this regard, we take a subsample from 2016 to 2020 to exclude the intervention of factors such as systemic risks and economic cycles. The empirical results are shown in Panel B of Table 5. We see that the regression coefficients for INGAPi,t2 and EXGAPi,t2 are all significantly positive, suggesting the conclusion are consistent with previous findings.

Third, add new control variables. In order to alleviate the impact of missing variables, following Habib et al. (2018), we introduce the corporate governance variables such as DUAL, defined as a dummy variable that equals 1 if f the CEO also holds the position of the chair of the board and 0 otherwise; DDR, defined as the ratio of the number of independent directors over the total number of directors on the board; BIG4, defined as a dummy variable that equals 1 if the firm employs Big Four auditors and 0 otherwise; FIRST_HOLD, defined as the percentage of shares held by the largest shareholder. The empirical results are shown in Panel C of Table 5. After controlling these factors, the regression coefficients of INGAPi,t2 and EXGAPi,t2 are all significantly positive, and reached a significance level of at least 5%. The results have not changed substantially. In general, the conclusions of this paper are robust and reliable.

## 5. Expansive research

### 5.1. The pay gap, property right and stock price crash risk

Considering the dual ownership structure of Chinese corporates, the U-shaped relationship between the pay gap of executives and future crash risk may be heterogeneous due to the different nature of property rights. First, there is a serious tendency of “official position thinking” in Chinese traditional culture (He et al., 2021). So, in addition to compensation incentives, state-owned corporate executives with Quasi-official status also have political promotion incentives (Cao et al., 2019). We can speculate that state-owned executives are relatively less sensitive to compensation. Second, an important way for executives to receive higher compensation is to improve corporate performance. Compared with non-state-owned executives, top executives of state-owned enterprises must assume more policy goals and social responsibilities, and their salaries are generally set by government departments. This results in a relatively low pay-performance sensitivity (Li et al., 2014). Third, senior executives of state-owned enterprises have a dual need for salary increase and political promotion. Due to political prospects and reputation considerations, they are inclined to reduce Short-sighted behaviors to avoid staining their political careers. Given all this, we predict that the above U-shaped relationship in state-owned corporate is not as obvious as that of non-state-owned corporate.

In refer to Huang and Liu (2021), we re-perform our regression analysis after partitioning the sample based on the property right, and report the results in this table. The results in columns (1)–(4) of Table 6 show that for the internal compensation gap, the regression coefficients of INGAP_i,t_ and INGAPi,t2 are significantly positive and negative, respectively in the non-state-owned group. In the state-owned group, the regression coefficients of INGAP_i,t_, INGAPi,t2 are not significant, as shown in columns (5)–(8) of Table 6. The U-shaped relationship between internal compensation gap and future crash risk is mainly reflected in non-state-owned corporates. For the external compensation gap, the regression coefficients of EXGAP_i,t_ are significantly negative in both groups, and the regression coefficients of EXGAPi,t2 are significantly positive. However, the regression coefficients of EXGAPi,t2 in non-state-owned corporates are significantly larger than those in state-owned corporates. That is to say that non-state-owned corporates have higher vertex curvature.^5^ The result suggests that although there is a U-shaped relationship between the external compensation gap and future crash risk, this relation may be more pronounced in non-state-owned corporates. The above inference is verified.

**TABLE 6:** The pay gap and crash risk: Effects of property right.

	State-owned		Non-state-owned		State-owned		Non-state-owned	
	NCSKEW_i,t+ 1_	DUVOL_i,t+ 1_	NCSKEW_i,t+ 1_	DUVOL_i,t+ 1_	NCSKEW_i,t+ 1_	DUVOL_i,t+ 1_	NCSKEW_i,t+ 1_	DUVOL_i,t+ 1_
								
	(1)	(2)	(3)	(4)	(5)	(6)	(7)	(8)
INGAP_i,t_	–0.0011	–0.0010	–0.0051**	–0.0034**				
	(–0.34)	(–0.49)	(–2.37)	(–2.39)				
INGAPi,t2	0.0001	0.0000	0.0003***	0.0001**				
	(0.72)	(0.38)	(2.91)	(2.55)				
EXGAP_i,t_					–0.0380	–0.0151	–0.0497	–0.0420*
					(–1.41)	(–0.85)	(–1.53)	(–1.95)
EXGAPi,t2					0.0174**	0.0105**	0.0198**	0.0183***
					(2.42)	(2.13)	(2.18)	(3.00)
Controls	Yes	Yes	Yes	Yes	Yes	Yes	Yes	Yes
Constant	–0.7285	–0.1053	–1.1388***	–0.4006	–1.1388***	–0.4006	–0.6965	–0.0806
	(–1.13)	(–0.23)	(–2.66)	(–1.31)	(–2.66)	(–1.31)	(–1.07)	(–0.17)
YEAR_i,t_/IND_i,t_	Control	Control	Control	Control	Control	Control	Control	Control
*N*	10,308	10,308	17,862	17,862	17,862	17,862	10,308	10,308
*R* ^2^	0.055	0.059	0.057	0.054	0.057	0.054	0.056	0.060

This table presents the results of the subsample analysis on the impact of the impact of the executive pay gap on stock price crash risk, and contains the results for both SOEs and non-SOEs. The t-statistics reported in parentheses are based on standard errors clustered by both firm and time. To save space, we omit the coefficient estimates of the control variables. Year and industry fixed effects are included. Here *, **, and ***Indicate statistical significance at the 10, 5, and 1% levels, respectively.

### 5.2. The pay gap, compensation fairness and stock price crash risk

Drawing on social comparison theory, executives often compare their input and outcome tradeoffs with a reference point to judge the fairness of their compensation (Ridge et al., 2015). However, with the increasing transparency of compensation data, the selection of reference point is not unique. In addition to core managers and same-level managers (Henderson and Fredrickson, 2001; Ridge et al., 2015), peer-reviewed salary benchmark is also an important reference for their comparison (Faulkender and Yang, 2010). We deem that there may be a “complementary” relationship between the external (internal) compensation fairness and the internal (external) compensation gap. Taking INGAP as an example, when managers feel the external compensation fairness, even if the INGAP is unreasonable, the tolerance will also increase. However, when managers feel external compensation unfairness, even if the INGAP is more reasonable, the satisfaction will also decrease. In other word, when executives feel the pay fairness, their sensitivity to the pay gap will be reduced, which is mainly manifested in weakening incentives and increasing tolerance for pay gap. It can be inferred that the compensation fairness is likely to affect the relationship between the pay gap and future crash risk.

According to the degree of internal and external compensation fairness, we re-perform our regression analysis after partitioning the sample based on the median values of compensation fairness, and report the results in this table. Among them, if the internal compensation gap is less than the median values of internal compensation gap in the same industry, it is regarded as a high internal compensation fairness group, otherwise it is low group. In the same way, if the external compensation gap is greater than the median values of external compensation gap in the same industry, it is regarded as a high external compensation fairness group, otherwise it is low group. The results are presented in Table 7. For the internal compensation gap, columns (1)–(4) show that the regression coefficients of INGAPi,t2 are in high, external fairness group lower than those in low external fairness group. In the low external fairness group, the result as shown in columns (5)–(8) is consistent with the above. Hence, the findings suggest compensation fairness mitigates the impact of the compensation gap on future crash risk.

**TABLE 7:** The pay gap and crash risk: Effects of compensation fairness.

	High external fairness group		Low external fairness group		High internal fairness group		Low internal fairness group	
	NCSKEW_i,t+1_	DUVOL_i,t+1_	NCSKEW_i,t+1_	DUVOL_i,t+1_	NCSKEW_i,t+1_	DUVOL_i,t+1_	NCSKEW_i,t+1_	DUVOL_i,t+1_
				
	(1)	(2)	(3)	(4)	(5)	(6)	(7)	(8)
INGAP_i,t_	–0.0027	–0.0026	–0.0075***	–0.0037**				
	(–1.07)	(–1.62)	(–2.67)	(–2.00)				
INGAPi,t2	0.0002*	0.0001*	0.0003**	0.0002*				
	(1.72)	(1.95)	(2.18)	(1.95)				
EXGAP_i,t_					–0.017	–0.018	–0.066**	–0.038*
					(–0.60)	(–0.93)	(–1.98)	(–1.73)
EXGAPi,t2					0.011	0.011**	0.030***	0.019***
					(1.32)	(2.08)	(3.36)	(3.28)
Controls	Yes	Yes	Yes	Yes	Yes	Yes	Yes	Yes
Constant	–0.4066	–0.0573	–1.0838**	–0.4292	–0.655	–0.081	0.410	0.417
	(–0.66)	(–0.15)	(–2.17)	(–1.35)	(–1.50)	(–0.29)	(0.40)	(0.59)
YEAR_i,t_/IND_i,t_	Control	Control	Control	Control	Control	Control	Control	Control
*N*	14,378	14,378	14,127	14,127	14,973	14,973	13,532	13,532
*R* ^2^	0.047	0.047	0.072	0.068	0.055	0.054	0.060	0.059

This table presents the results of the subsample analysis on the impact of the impact of the executive pay gap on stock price crash risk. The high compensation fairness subsample includes firm-years with above-median compensation fairness, and the low compensation fairness subsample includes firm-years with below-median compensation fairness. To save space, we omit the coefficient estimates of the control variables. The t-statistics reported in parentheses are based on standard errors clustered by both firm and time. Year and industry fixed effects are included. Here *, **, and ***Indicate statistical significance at the 10, 5, and 1% levels, respectively.

### 5.3. Mediating mechanism analysis

Our research shows that there is a U-shaped relationship between the compensation gap and future crash risk. But the transmission path behind it has not been verified. As described above, the bad news hoarding theory is an important factor to explain stock price crash risk. Senior managers are motivated and capable to use power and resources to withhold and accumulate negative information, in order to maximize personal benefits. This inevitably leads to a decline in the quality of information disclosure. Many scholars provide similar empirical evidence (Hutton et al., 2009; An et al., 2020). We can infer that the quality of information disclosure may be an important transmission path for compensation gap to affect future crash risk.

In view of the non-linear relationship between variables, the use of the “three-step” method of mediation effect developed by Baron and Kenny (1986) cannot effectively test the mediation effect of the quality of information disclosure. In this regard, we adopt the moderated path analysis of Edwards and Lambert (2007) to test the hypotheses proposed. This method can fully analyze the mediation effects on all possible paths in the mediation model, thereby revealing the mediation effects between independent and dependent variables (Edwards and Lambert, 2007). Specifically, this analysis mainly includes two general regression equations:


(5) $$\text{Y}=\alpha_{1}+\alpha_{2}\,\text{X}+\alpha_{3}\,\text{M}+\alpha_{4}\,\text{Z}+\alpha_{5}\,\text{XZ}+\alpha_{6}\,\text{MZ}+\varepsilon_{1}$$

(6) $$\text{M}=\alpha_{1}+\alpha_{2}\,\text{X}+\alpha_{3}\,\text{Z}+\alpha_{4}\,\text{XZ}+\varepsilon_{2}$$

Where, Y is dependent variable, risk of stock price crash. X is independent variable, compensation gap. M is mediating variable, quality of information disclosure. Z is moderating variable, compensation gap. XZ is the square term of the compensation gap. MZ is the interaction term of information disclosure quality and compensation gap. ε_*1*_ and ε_*2*_ are residuals. Equation (5) is used to test the U-shaped relationship between the pay gap and stock price crash risk and the mediating role of information disclosure quality. Equation (6) is used to test the inverted U-curve effect of compensation gap on the quality of information disclosure.

We take the information disclosure evaluation results of listed firms published by Shenzhen Stock Exchange as the proxy indicator of the quality of information disclosure (INFQ_i,t_). Based on the evaluation results, we assign INFQ_i,t_ in order: 4-excellent (A), 3-good (B), 2-qualified (C), 1-disqualification (D). The control variables of Equation (5) are consistent with Equation (4). We include a series of control variables in Equation (6): ROA, SIZE, LEV, MB, INST, CEO_SHARE, INST. We also include industry and year dummies to control for industry and time FEs, respectively.

The regression results are shown in Table 8. Taking the INGAP as an example, column (3) shows that the regression coefficients of INGAP_i,t_ and INGAPi,t2 are significantly positive and negative, respectively. The results show that there is an inverted U-shaped relationship between internal compensation and gap quality of information disclosure. Columns (1) and (2) show that the regression coefficients of INGAPi,t2 are significantly positive, which further verifies hypothesis 1a. The regression coefficients of INFQ_i,t_ are both significantly positive at the 5% level, confirming the bad news hoarding theory. The regression coefficients of the interaction terms INGAP_i,t_×INFQ_i,t_ are not significant, which means that the relationship between the quality of information disclosure and the risk of stock price crash is not affected by the INGAP. Judging from this, the quality of information disclosure plays a mediation effect in the U-shaped relationship between the INGAP and the risk of stock price crash. The empirical results of columns (4)–(6) are similar to those of columns (1)–(3). We can know from that, the quality of information disclosure mediates the relationship between the external compensation gap and crash risk. In summary, the quality of information disclosure path has been confirmed.

**TABLE 8:** Mediation effect of information disclosure quality.

	Internal compensation gap			External compensation gap		
	NCSKEW_i,t+1_	DUVOL_i,t+1_	INFQ_i,t_	NCSKEW_i,t+1_	DUVOL_i,t+1_	INFQ_i,t_
						
	(1)	(2)	(3)	(4)	(5)	(6)
INGAP_i,t_	–0.0028	–0.0023*	0.0000**			
	(–1.38)	(–1.70)	(2.43)			
INGAPi,t2	0.0002**	0.0001*	–0.0001***			
	(2.08)	(1.89)	(–5.29)			
EXGAP_i,t_				–0.0606**	–0.0444***	0.0469***
				(–2.42)	(–2.68)	(11.94)
EXGAPi,t2				0.0228***	0.0189***	–0.0099***
				(3.21)	(3.95)	(–8.55)
INFQ_i,t_	–0.0262**	–0.0148**		–0.0256**	–0.0145**	
	(–2.50)	(–2.11)		(–2.45)	(–2.07)	
INGAP_i,t_×INFQ_i,t_	0.0003	–0.0000				
	(0.21)	(–0.00)				
EXGAP_i,t_×INFQ_i,t_				0.0001	–0.0010	
				(0.01)	(–0.12)	
Controls	Yes	Yes	Yes	Yes	Yes	Yes
Constant	–1.1124***	–0.2179	0.0521	–1.1546***	–0.2209	0.1683***
	(–2.95)	(–0.93)	(1.38)	(–3.00)	(–0.93)	(4.27)
YEAR_i,t_/IND_i,t_	Control	Control	Control	Control	Control	Control
*N*	21,163	21,163	21,163	21,163	21,163	21,163
*R* ^2^	0.056	0.056	0.009	0.057	0.056	0.009

This table presents the mediation results of pay gap of executives affecting stock price crash risk by using the fixed effect regression. The sample contains firm-years from 1995 to 2008 with non-missing values for all the control variables. As INFQ is measured as the information disclosure evaluation results of listed corporates published by Shenzhen Stock Exchange, the sample observations of the regression results have changed. Year and industry fixed effects are included. The t-statistics reported in parentheses are based on standard errors clustered by both firm and year. *, **, and ***Indicate statistical significance at the 10, 5, and 1% levels, respectively.

## 6. Conclusion

Frequent stock price crashes in recent years have aroused widespread concern in the entire society. As an important part of the compensation contract, whether the compensation gap promotes or suppresses future crash risk is an important empirical question to be explored. Using a sample of Chinese A-share listed firms over the period 2010–2020, we find the strong evidence that there is a U-shaped relationship between the internal and external compensation gaps of executives and crash risk. In other words, pay gap is negatively related to crash risk, but reaching an apex at moderate levels, it is positively related thereafter. After endogenous analysis and robustness testing, the results are still valid. In addition, the U-shaped relationship is mainly reflected in non-state-owned and low compensation fairness of firms. We also find that the quality of information disclosure plays a mediation effect in the U-shaped relationship.

Our findings have important practical implications. First, we confirmed that there is a U-shaped relationship between the executive pay gap and crash risk. When designing the compensation structure, on the one hand, the internal and external salary gap should be properly widened to ensure the competitiveness and incentive of the compensation structure. This is because the pay structure can promote the hard work of executives, ease the conflict between principals and agents, and curb executives short-sighted behavior. On the other hand, the rationality and fairness of compensation distribution should be fully considered, which contributes to avoiding the opportunistic behaviors of senior executives caused by the huge gap between internal and external compensation. In short, enterprises should try their best to set the value of the pay gap near the inflection point of the U-shaped curve, so as to ensure that the probability value of the company’s future stock price crash risk is the lowest.

Second, we find that the impact of compensation gap on crash risk varies depending on the nature of the property rights. In view of this, first, state-owned firms should design a competitive compensation structure to reduce future crash risk. State-owned enterprises need to adhere to the market-oriented reform direction, establish a scientific and reasonable performance evaluation system, and increase the ratio weight of company performance. Through these methods, the sensitivity of state-owned enterprise executives to the company’s pay gap is increased and healthy competition among executives is promoted. Second, for non-state-owned enterprises, it is necessary to improve the internal control system, refine the process, and further reduce the space for executives to use public power to seek private interests. At the same time, relevant regulatory authorities need to increase the penalties for executives’ bad news concealing, and try to increase the litigation risk and reputation cost of their salary manipulation as much as possible. External supervision and internal governance work along both lines, aimed at reducing opportunistic behaviors caused by the large pay gap in non-state-owned enterprises.

Finally, our research shows that pay fairness is an important factor influencing the relationship between executive pay gap and stock price crash risk. Therefore, when constructing the compensation system for senior management team, firms should coordinate the design of internal and external compensation gaps to prevent adverse selection and moral hazard caused by the compensation unfairness. Meanwhile, compensation fairness can also play a role of “complementary” and “replacement.” Firms can improve the compensation fairness and reduce the impact of excessive compensation gap on future crash risk. It is worth emphasizing that firms cannot blindly pursue compensation fairness and reduce pay incentives, and there is necessity to give full play to the combined effect of the two.

## Data availability statement

The raw data supporting the conclusions of this article will be made available by the authors, without undue reservation.

## Ethics statement

Ethical review and approval was not required for the study on human participants in accordance with the local legislation and institutional requirements. Written informed consent to participate in this study was provided by the participants’ legal guardian/next of kin.

## Author contributions

QL contributed to the conception of the study. ZP performed the data analyses and wrote the manuscript. Both authors contributed to the manuscript revision, read, and approved the submitted version.

## Footnotes

1 On March 21, 2017, *ST Kunji exposed the company’s major financial fraud scandal, the regulatory authorities confirmed that the company during 2013 to 2016, suspected of inflated profits, financial fraud, fabricated facts and other violations. Twenty-eight senior executives at the time were involved in the case. And, the company’s chairman, Wang Xing, was banned from the market for life. The survey found that the average executive team was paid just 243,000, less than half the average of their peers. After that, the company appeared 6 consecutive daily limits, and indirectly led to delisting.

2 According to different reference objects, the pay gap of executives can be subdivided into the internal pay gap and the external pay gap. Among them, the internal pay gap of executives refers to the pay gap between core and non-core executives. The external compensation gap of executives refers to the pay gap between executive compensation and peers’ compensation benchmarks (same industry).

3 Panel data can be estimated by pooled regression model (POLS), fixed effect model (FE) and random effect model (RE). After the Hausman test and the LM test, we run the regression equations with firm fixed effect.

4 Drawing on Equation 4, the inflection point can be calculated as: x* = –γ1/2γ2.

5 The vertex curvature of quadratic function is the second-order reciprocal of the quadratic function. For a U-shaped curve, the larger the vertex curvature, the steeper the U-shaped curve. The smaller the vertex curvature, the smoother the U-shaped curve.
